# Supplementary material for: A Straightforward Approach to Synthesize 7-Aminocephalosporanic Acid In Vivo in the Cephalosporin C Producer Acremonium chrysogenum
Source: J Fungi (Basel). 2022 Apr 26;8(5):450. doi: 10.3390/jof8050450 (PMC9144927; doi:10.3390/jof8050450)
Supplement: Supplementary file 1 [file jof-08-00450-s001.zip › Lin et al. Supplementary File 1.pdf]

Supplementary File 1

Table S1. Oligonucleotides used in this study.

Oligonucleotide	Sequence (5´→3´)	Specificity
MCS_pAB	GCGGCCGCGAGACCGCTAGCTCGAGAATTCTACGTACTAGTAGATCTGGTCTCGGATCCA	Insertion of *Bsa*I cutting site forward
MCS_pAB-rev	TGGATCCGAGACCAGATCTACTAGTACGTAGAATTCTCGAGCTAGCGGTCTCGCGGCCGC	Insertion of *Bsa*I cutting site reverse
His-CCAA-ATGdel_F	GGTCTCAGCCGATGCACCACACCATCACCACGGCGGTGGTGGCTCAGAGCCGACATCAACCCCACAGGC	CCAA N terminal modification forward
CCAA_R	CACGGTCTCTGATCTCAGGCG	CCAA reverse
CCAA inter seq_F	ACTTCTTCAGAACCCCCACTGAG	CCAA sequencing forward-1
Opt-TrpC-pAB_F	TTGGTCTCGGTGCCACTTAACGTTACTGAAATCATCAAACAGC	GG PCR vector pAB-nat forward
pXUL-10_F-2	AACTGGGGTCTCCTGGTATGCACCACACCATCACCACG	EGFP-CCAA: GG PCR insert CCAA forward
pXUL-10_R-2	AACTGGGGTCTCCATCCGGCGTAGTCGGGGACGTCG	EGFP-CCAA: GG PCR insert CCAA reverse
CCAC_SF	AGACGTCTCGGGCTGCTGATG	CCAC sequencing forward
CCAC_SR	GCTGGGGACGAGGCCGTTGG	CCAC sequencing reverse
1751	GCCATATTTTCCTGCTCTCC	Pgpda sequencing forward
1757	AGCTGACATCGACACCAAG	TtrpC sequencing reverse
Southern probe_F	CTGCATACAAGCCACGCTCG	SB probe CCAA forward
Southern probe_R	CGGAGCAACGCCGTTGAAC	SB probe CCAA reverse
SB_a-tubulin_F	GGCTGGTTGCCAGATTGC	SB probe a-tubulin forward
SB_a-tubulin_R	GAGTTGGCCTCGTGAGC	SB probe a-tubulin reverse
CCAA-int seq_F2	GCTGAGTACTGGGGCCCGGATTATG	CCAA sequencing forward-2
CCAA-int seq_R	GAGGATCATCCGGGAGGCGTCG	CCAA sequencing reverse

Table S2. Agilent 1100 HPLC gradient elution method for measuring beta-lactam substances[1].

Time (min)	0	3	4	8	9	10	10.25	12
Eluent A (%)	100	100	95	90	5	5	100	100
Eluent B (%)	0	0	5	10	95	95	0	0

[1]Eluent A: 100% H2O+0.05% H_3PO_4; Eluent B: 90% ACN+0.05% H_3PO_4; Flow rate: 1.2 ml/min; Elution time: 12 min; Sample injection volume: 2 µl; Sample storage: 4°C, Column temperature: 40°C

A

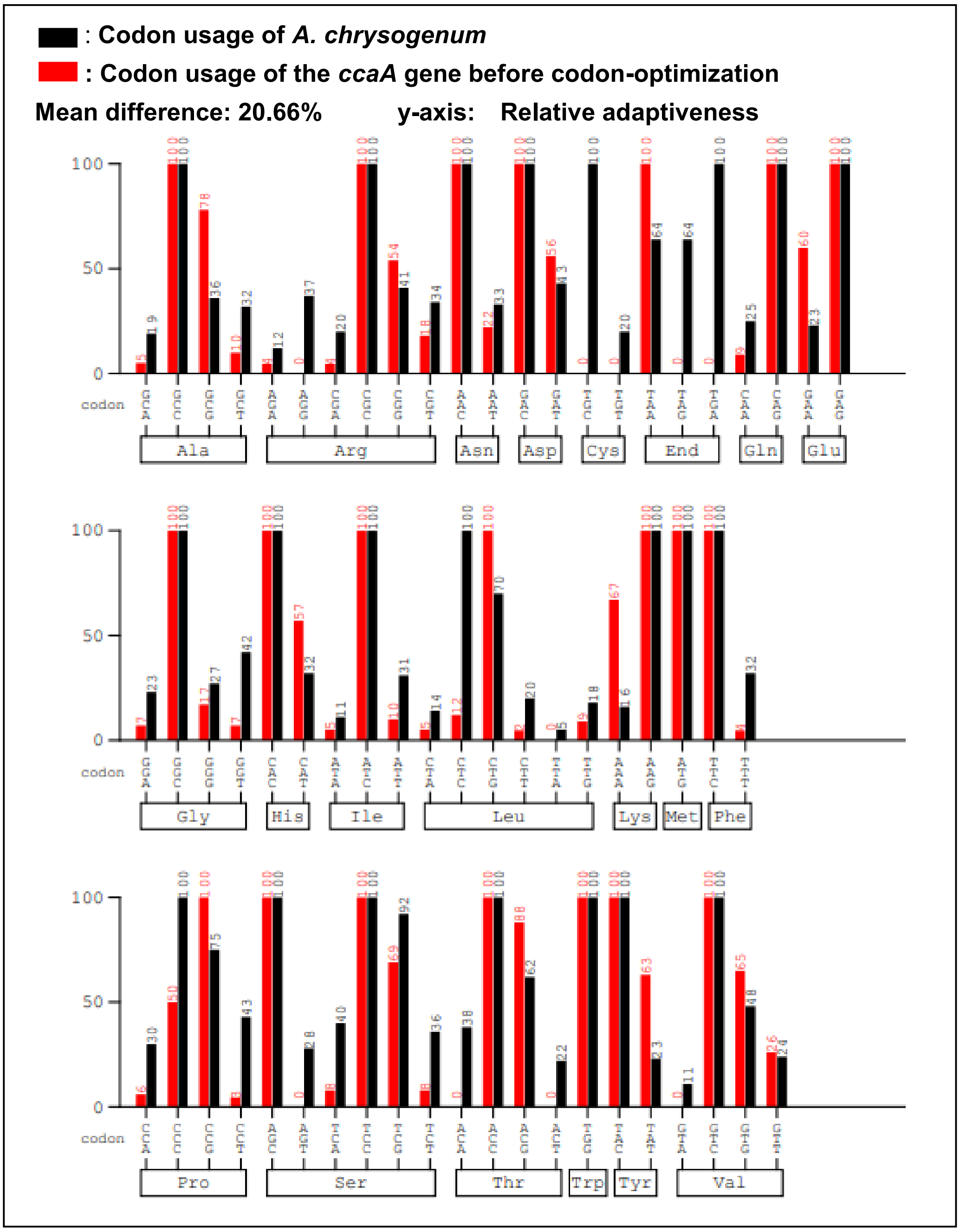
: Codon usage of *A. chrysogenum*
: Codon usage of the *ccaA* gene before codon-optimization
Mean difference: 20.66%
y-axis: Relative adaptiveness
100
50
0
codon
Ala
Arg
Asn
Asp
Cys
End
Gln
Glu
Gly
His
Ile
Leu
Lys
Met
Phe
Pro
Ser
Thr
Trp
Tyr
Val

B

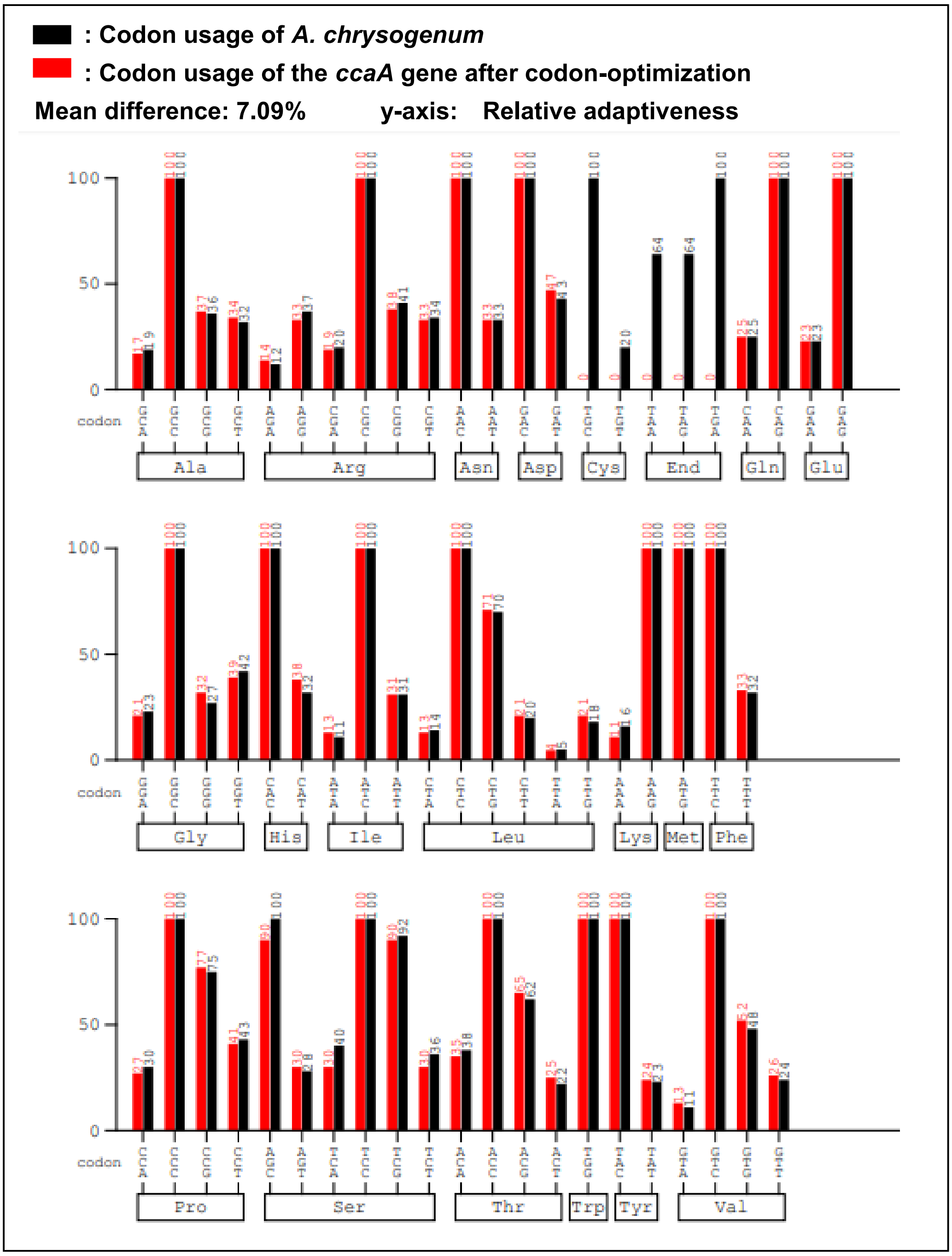
: Codon usage of A. chrysogenum
: Codon usage of the ccaA gene after codon-optimization
Mean difference: 7.09%
y-axis: Relative adaptiveness
codon
Ala
Arg
Asn
Asp
Cys
End
Gln
Glu
Gly
His
Ile
Leu
Lys
Met
Phe
Pro
Ser
Thr
Trp
Tyr
Val

C

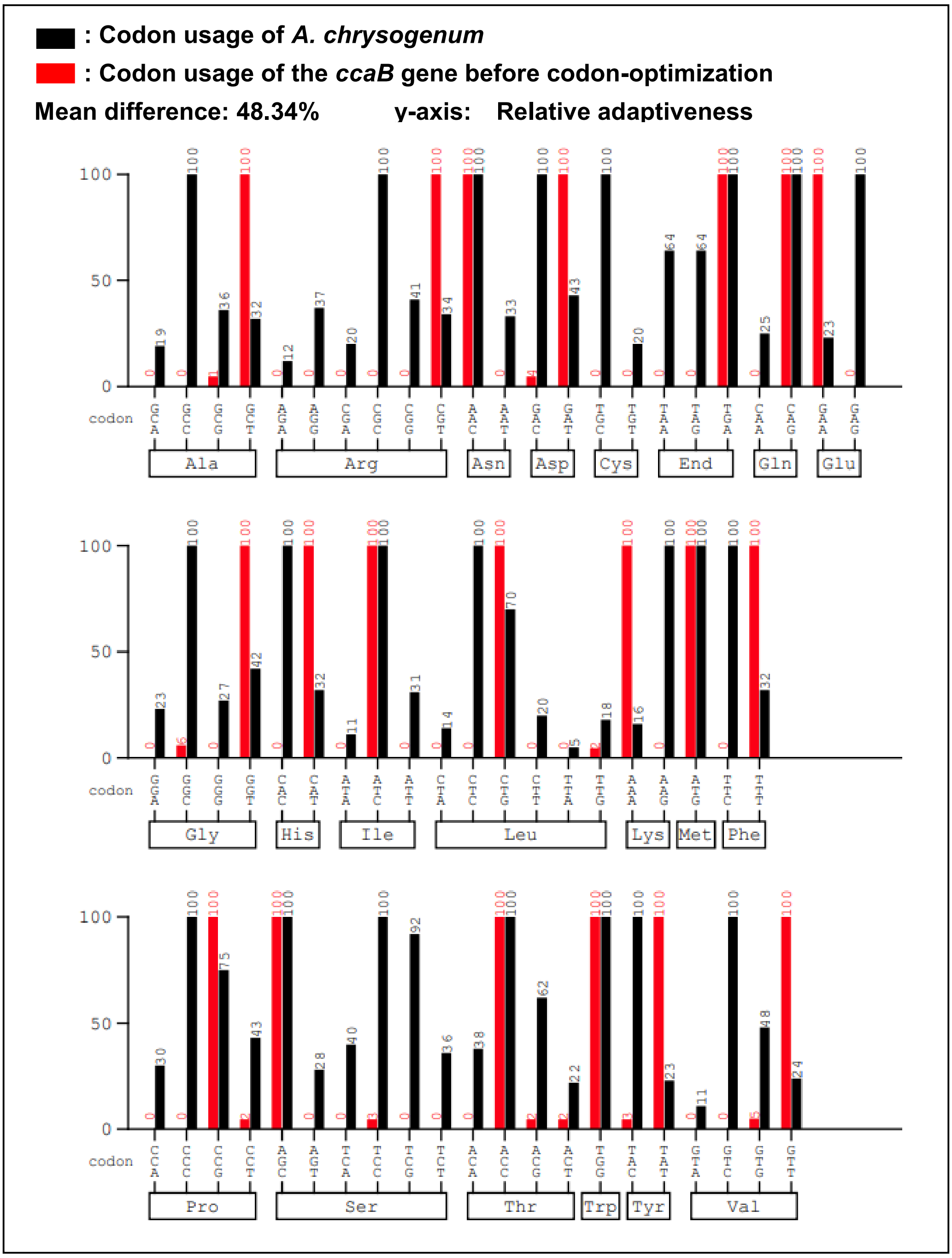
: Codon usage of A. chrysogenum
: Codon usage of the ccaB gene before codon-optimization
Mean difference: 48.34%
y-axis: Relative adaptiveness
codon
Ala
Arg
Asn
Asp
Cys
End
Gln
Glu
Gly
His
Ile
Leu
Lys
Met
Phe
Pro
Ser
Thr
Trp
Tyr
Val

D

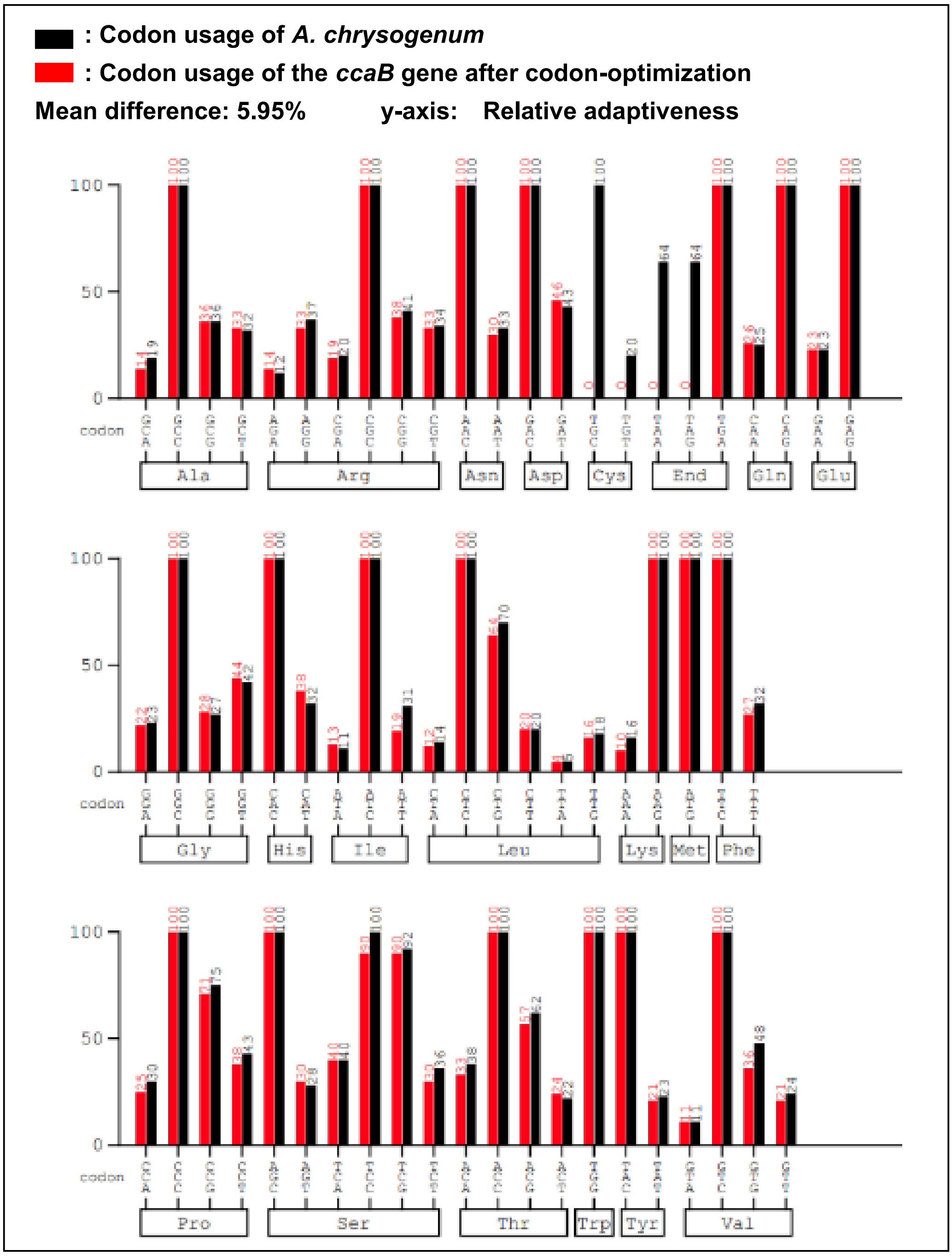

Figure S1. Codon optimization of the CCA coding sequences. (A) Codon usage bias between the investigated organism *A. chrysogenum* and the *ccaA* gene sequence originated from *Pseudomonas sp. GK16*. (B) Codon usage comparison of *ccaA* after codon-optimization. (C) Codon usage bias between *A. chrysogenum* and the *ccaB* sequence from *Pseudomonas sp. GK16*. (D) Codon usage comparison of *ccaB* after codon-optimization.

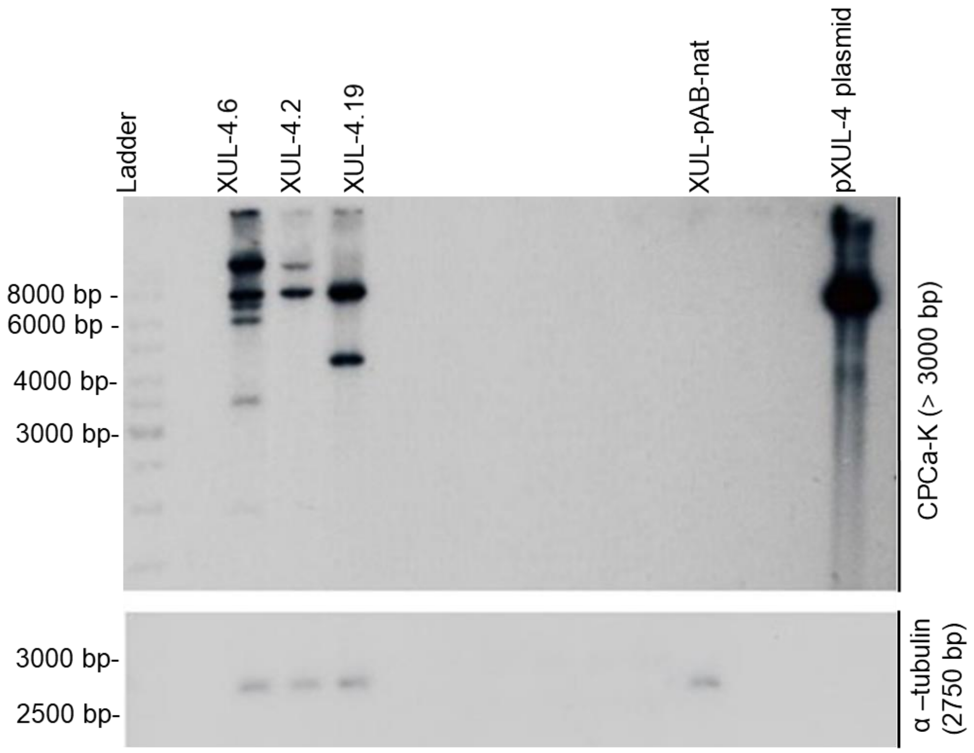

Figure S2. Southern blot analysis of recombinant CCA transformants to assess the copy number of gene integration. 30 µg of genomic DNA of recombinant CCAA transformants were digested with *Hind*III. Southern blot hybridization was performed with a radioactively 32P-labeled probe specifically detecting the *ccaA* gene. For the quantification of the gene copy number, a labeled probe detecting α-tubulin coding gene was used as a reference. For the quantification of the *ccaA* gene copy number, the band with the weakest signal intensity, which is the 3500 bp signal from XUL-4.6, is defined as one copy of the *ccaA* gene.

A

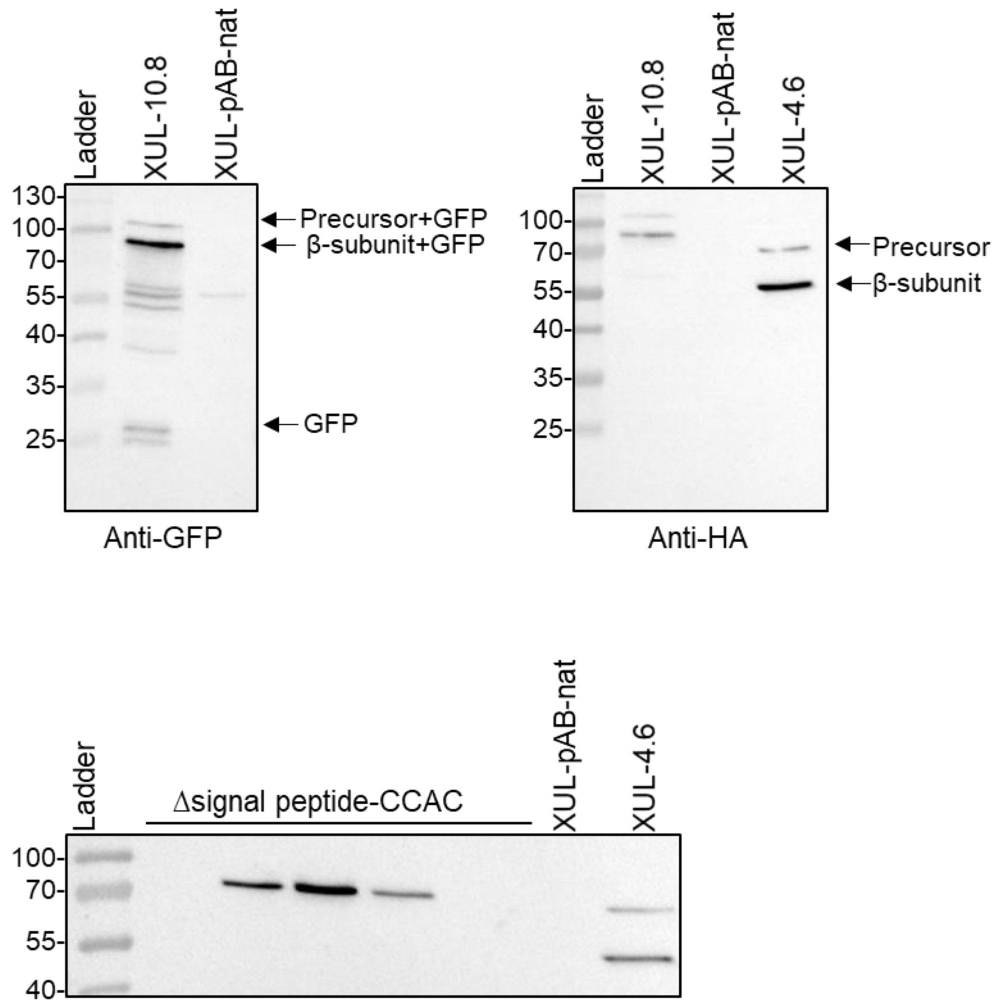

Figure S3. Supplementary western blot results. (A) Detection of C-terminal EGFP fused CCAA transformant (XUL-10.8) using anti-GFP antibody and anti-HA antibody. (B) Precursor bands detected from signal peptide deleted CCAC transformants.

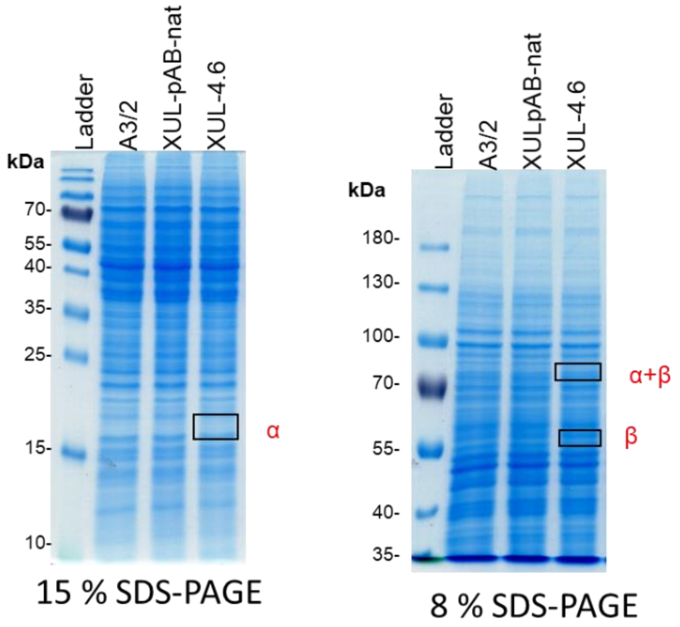

Figure S4. Example of preparing samples for protein MS analysis. SDS-PAGE was performed with 8% and 15% of gels followed by coomassie staining was followed the protein separation. The gel slices at the expected locations for the precursor, α-subunit, and β-subunit were cut out for enzyme digestion and further sample preparations.

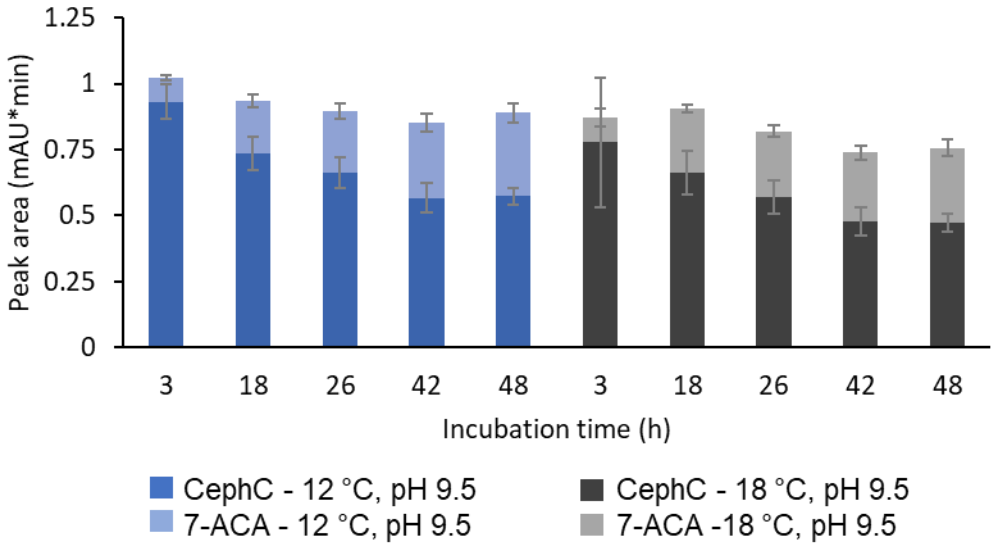

Figure S5. Time- and temperature-dependent degradation of CCA substrate and product. Shown are results for the XUL-4.6 strain, which was grown under conditions as described in the legend of Fig. 8A. HPLC samples were taken from the supernatants (pH 9.5) at different time points of incubation at 12°C (blue bars) or 18°C (grey bars). The peak areas of 7-ACA and CPC are presented as a cumulative bar chart.

A

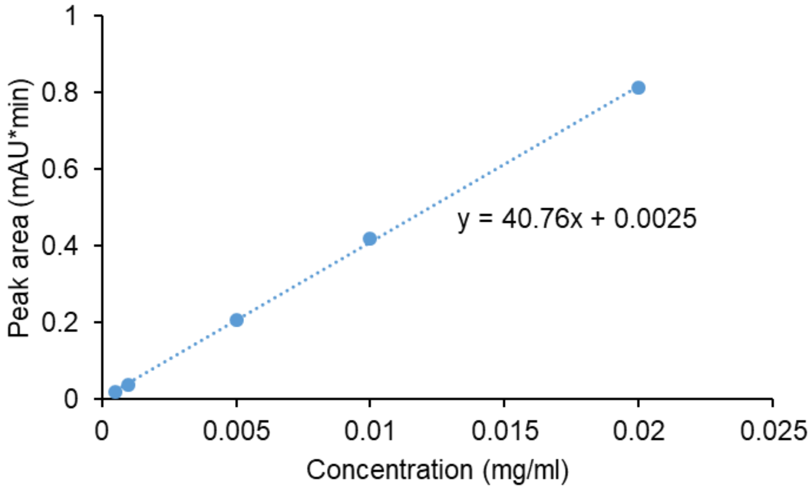

B

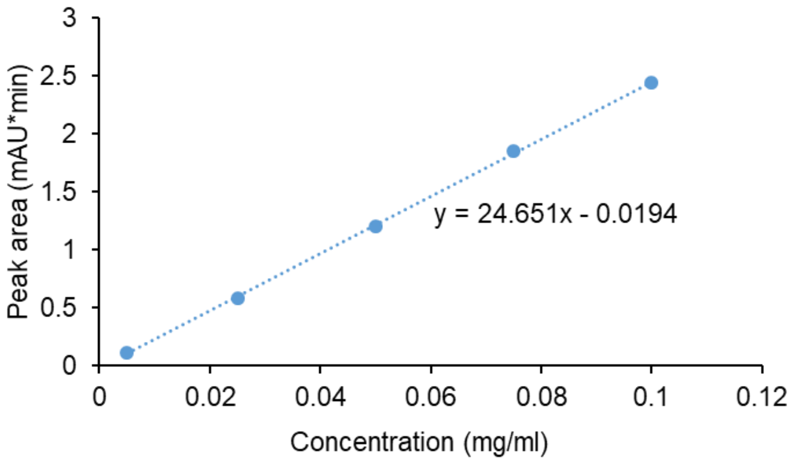

Figure S6. HPLC calibration curves generated using standard substances. The series dilutions of standard substances were prepared and measured by the identical method used for measuring all other samples. (A) The calibration curve of 7-ACA. (B) The calibration curve of CPC.

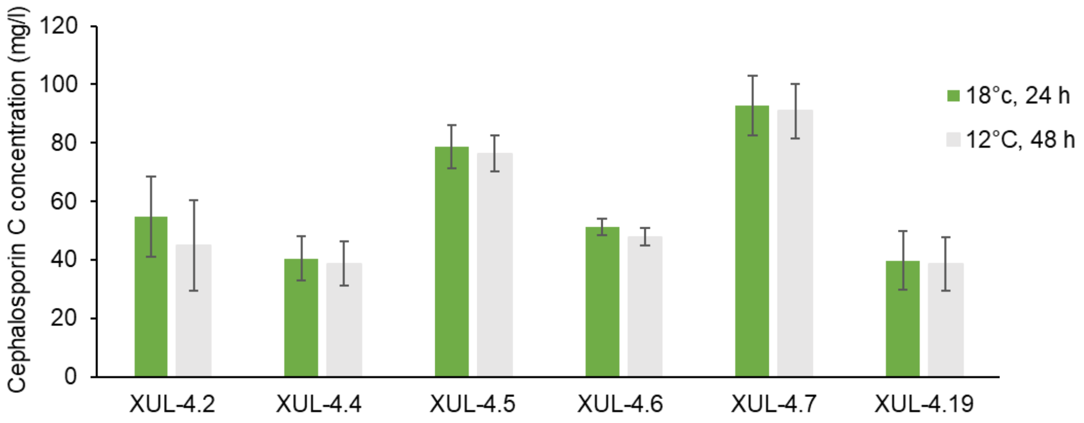

Figure S7. Investigation of CPC productivity of CCA transformants. Six out of 19 analyzed CCAA transformants are shown, and the pH of the culture supernatants from 72 hours of liquid culture was adjusted to 9.5. After harvesting supernatants, all samples were incubated for 24 hours at 18°C or 48 hours at 12°C. CPC concentrations of CCAA transformants were calculated according to the calibration curve generated from HPLC measurement.

A

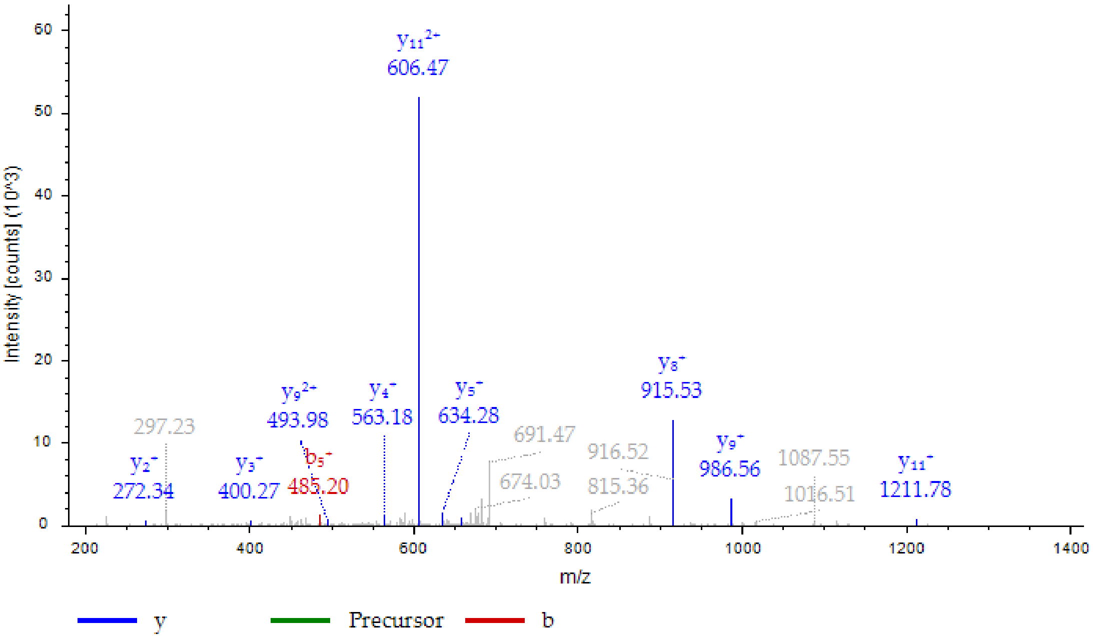

B

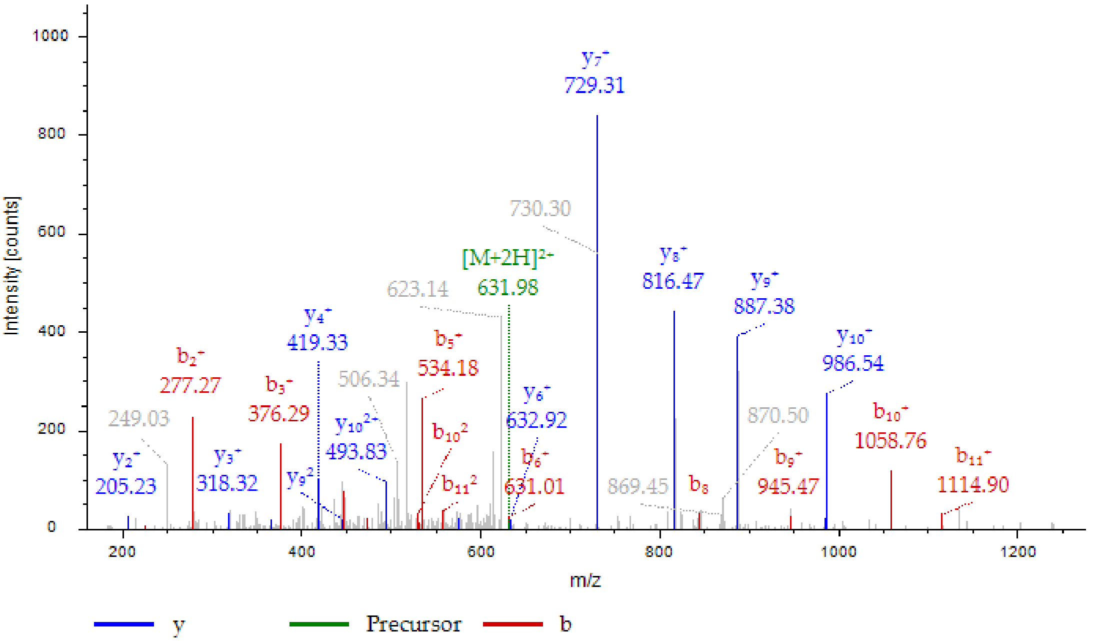

Figure S8. MS/MS-spectra of selected peptides. (A) Truncated N-Terminal peptide of the α-subunit. Sequence: STPQAPIAAYKPR, Charge: +2, Monoisotopic m/z: 700.38656 Da (-1.7 mmu/-2.42 ppm), MH+: 1399.76584 Da, RT: 32.6754 min, identified with: Sequest HT (v1.17); XCorr:2.39, Fragment match tolerance used for search: 0.8 Da. (B) C-terminal peptide of the α-subunit up to the autocatalytic site at AA171. Sequence: LYVASPGRTLGE, Charge: +2, Monoisotopic m/z: 631.84272 Da (+2.12 mmu/+3.36 ppm), MH+: 1262.67816 Da, RT: 34.3149 min, identified with: Sequest HT (v1.17); XCorr:3.44, fragment match tolerance: 0.8 Da.
